# Sternotomy Approach to the Anterior Cervicothoracic Spine

**DOI:** 10.7759/cureus.19421

**Published:** 2021-11-09

**Authors:** Brian Fiani, Daniel Chacon, Claudia Covarrubias, Erika Sarno, Athanasios Kondilis

**Affiliations:** 1 Neurosurgery, Desert Regional Medical Center, Palm Springs, USA; 2 Medicine, Ross University School of Medicine, Bridgetown, BRB; 3 Medicine, Universidad Anáhuac Querétaro, Santiago de Querétaro, MEX; 4 Medicine, Michigan State University College of Osteopathic Medicine, East Lansing, USA

**Keywords:** transsternal, partial sternotomy, manubriotomy, peribrachiocephalic, transthoracic

## Abstract

The anterior cervicothoracic spine is a challenging region to approach given the various vascular, osseous, nervous, and articular structures, which prevent adequate exposure. This region is susceptible to lesions ranging from tumors, degenerative disease, infectious processes, and traumatic fractures. Our objective was to critically evaluate the sternotomy approach in spine surgery to give the technical implications of its usage. The safety and efficacy of the transsternal approach are discussed as well as the advantages, disadvantages, indications, and contraindications. The transsternal approach is the most direct access to pathologies in the upper anterior cervicothoracic spine and enables the spine surgeon to gain direct exposure to the cervicothoracic junction for ideal visualization. Anatomical considerations must be kept in mind while performing a sternotomy to prevent complications such as denervation or bleeding. This technique is useful for the armamentarium of spinal surgeons.

## Introduction

The anterior cervicothoracic spine is a difficult region of the spine to approach because it contains various vascular, osseous, nervous, and articular structures that prevent adequate exposure [1-3]. The cervicothoracic spine is susceptible to lesions ranging from neoplasms, degenerative disease, infectious processes, and traumatic fractures [3-5]. The anterior surgical approaches including transthoracic, peribrachiocephalic, manubriotomy with clavicle resection, and partial sternotomy, have been developed and often offer the best management strategy for various anterior cervicothoracic pathologies [1,4,6,7]. There have been many modifications to this approach mainly to counterbalance and limit the extension of the osteotomy. Sternotomy, although mostly used in open-heart surgery, has evolved as a feasible spinal surgery approach that allows visualization of the anterior spine [8].

The anterior approach to the spine was first pioneered and directed for the treatment of Pott’s disease, dating back to 1894. In 1957, Cauchoix and Binet were the first to attempt an anterior approach through a direct median sternotomy for treating cervicothoracic spinal lesions and the surgical modality has continued to evolve [2,4,7]. Currently, the anterior transsternal approach is recommended by most surgeons due to the reported advantages of safety and feasibility [2]. This extensive technical report aims to examine current clinical and biomechanical evidence on the sternotomy approach to the spine. Herein, we summarize current evidence and discuss pertinent topics for the spinal surgeon considering this evolving approach, including indications, advantages, relevant anatomy, contraindications, and technical considerations.

## Technical report

Advantages

The anterior transsternal or median sternotomy approach is a feasible surgical option that provides direct access to cervicothoracic pathology, allowing for direct exposure of the anterior vertebral elements. With meticulous dissecting of the vascular compartment of the superior mediastinum, the many vital neurovascular structures close by can be directly visualized [1,4]. A full median sternotomy, with the incision being able to extend both cranially and caudally, is a technically less expansive procedure when compared to the other modified versions, avoiding clavicle resection and thus preserving the pectoral girdle [1]. Additionally, the inside or outside window of the brachiocephalic vasculature grants enhanced maneuverability for the reconstruction and stabilization of the four upper thoracic levels [6].

Some surgeons have proposed that the anterior approach via a modified transclavicular-transmanubrial approach with preservation of the sternoclavicular joint is better suited biomechanically for exploring the pathology of the cervicothoracic spine and its decompression and stabilization, granting better neurological outcome with good upper limb stability [9]. Overall, anterior approaches for anterior pathology have been reported to be more feasible than posterior or lateral approaches to anterior pathology, addressing the pathology directly and adequately with reduced operation time, transfusion requirements, early ambulation, maintaining alignment without deformity, and overall decreased injury to paraspinal structures [4,9].

Anatomic considerations

**Figure 1:**
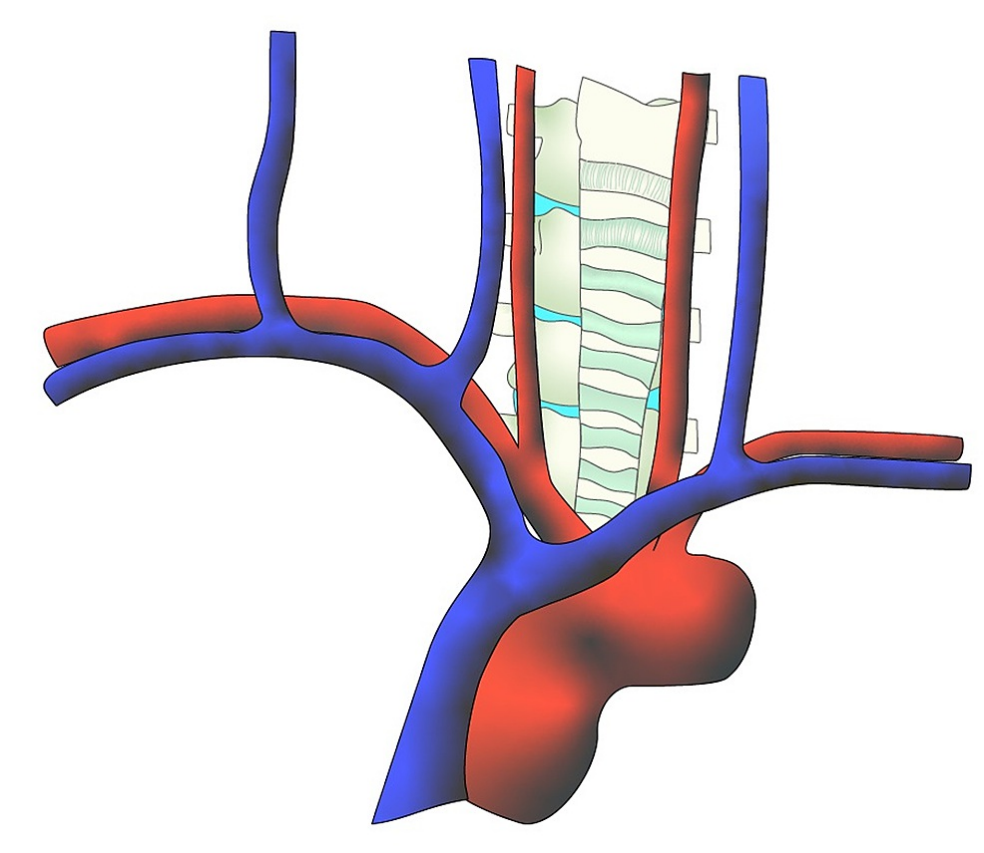
Standard anterior view of anatomy with aortic arch, subclavian veins with jugular venous arches, and trachea obstructing access to the spine. [Courtesy of Roger Avila]

A midline approach and incision to the sternum reveal many anatomical structures that lie anterior to the spine. Anatomical considerations including vasculature and nerves while performing a sternotomy must be paid close attention to in order to avoid subsequent complications including bleeding and denervation (Figure 1).

Vasculature

Avoidance of bleeding during a sternotomy can be achieved through careful maneuvering through the vasculature of the sternum. The manubrium and xiphoid process are two landmarks that allow for planning of the sternotomy incision at the superior and inferior end [10]. Immediately superior to the manubrium lies the jugular venous arch; made up by the connection of the bilateral anterior jugular veins [10,11]. Meanwhile, the inferior landmark made up by the xiphoid process contains another venous arch made up of the bilateral internal thoracic veins that connect transversely at the xiphisternal joint [10,12]. It is recommended to locate and clip both of these vessels to avoid excessive bleeding [10]. The body of the sternum is perfused by the perforating branches of the internal thoracic artery (ITA) bilaterally [13]. As the oscillating saw traverses the sternum during a median sternotomy, bleeding from the ITA can be controlled by cautery or the use of bone wax [10]. However, excess cauterization must be avoided as it can lead to necrosis and an increased risk of infection [10].

**Figure 2:**
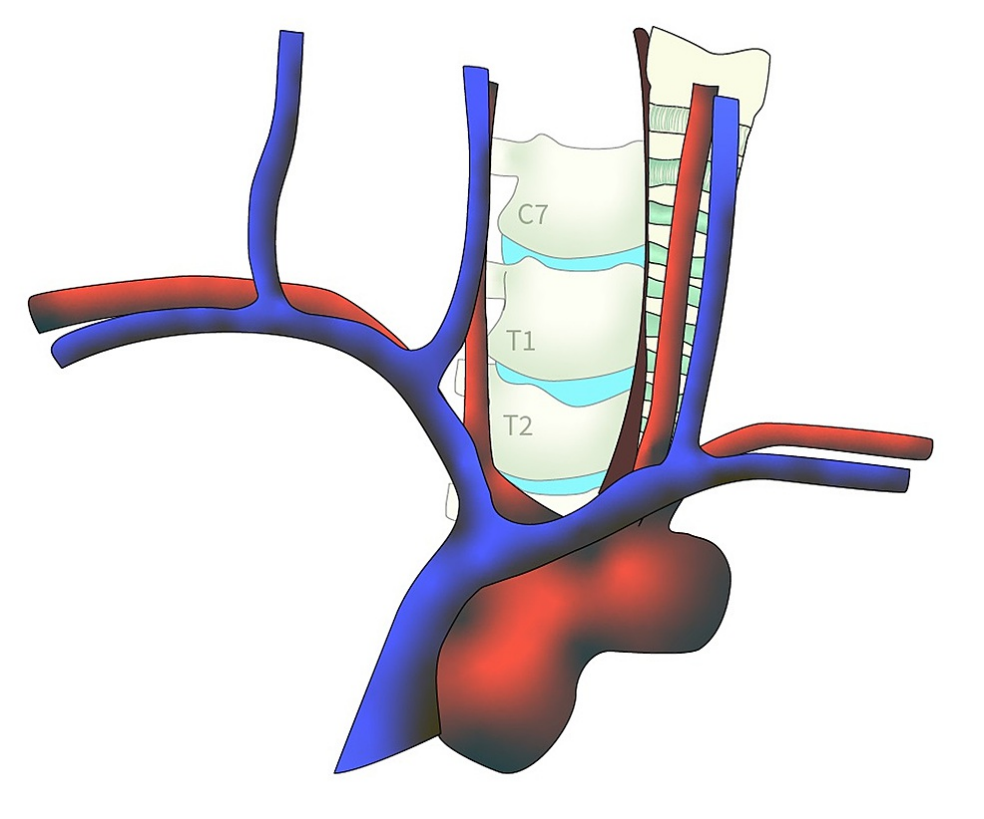
Inside window exposure. [Courtesy of Roger Avila]

**Figure 3:**
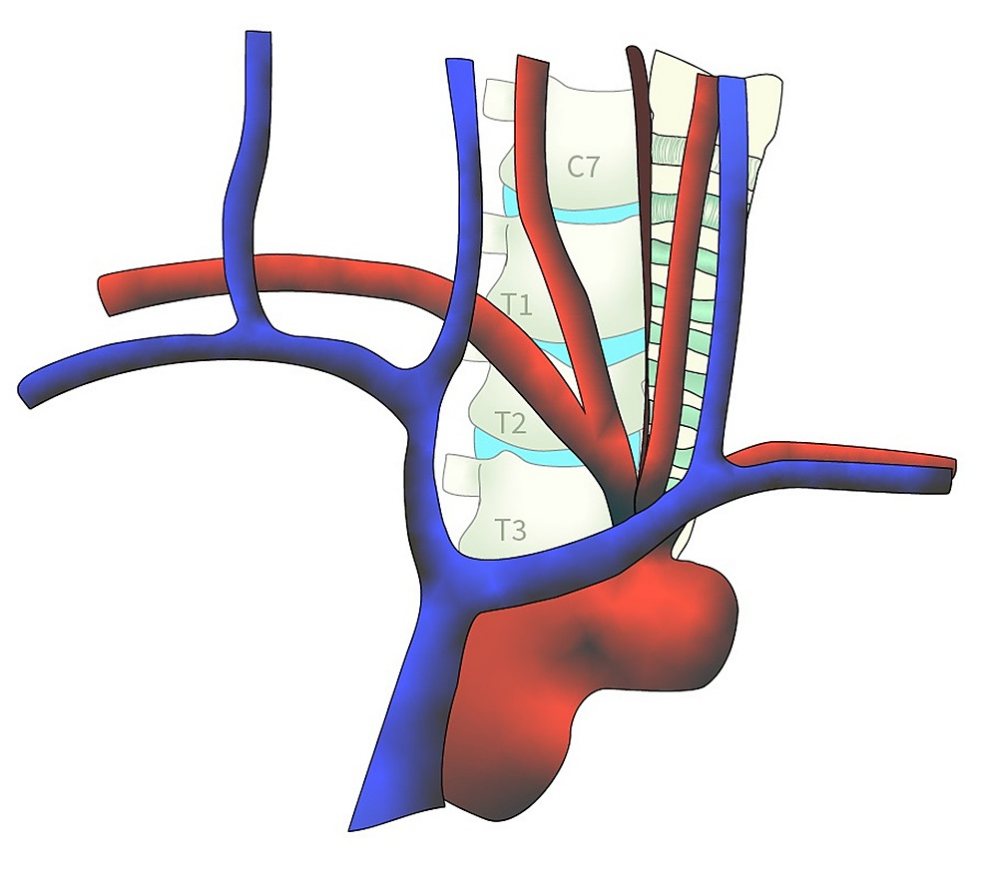
Outside window exposure. [Courtesy of Roger Avila]

While approaching the cervicothoracic vertebral bodies, access can be gained through windows created after manipulation of the brachiocephalic vessels and nearby vital structures [1,2,4]. The T1 and T2 vertebral bodies can be exposed through manipulation of the right brachiocephalic and common carotid artery, along with the trachea and esophagus [1,2,4]. The inside window is accessed through retraction of the right brachiocephalic and common carotid artery to the right, while the trachea and esophagus are retracted to the left (Figure 2) [1,2,4]. This allows for adequate exposure to the cervicothoracic junction down to the T2 level [1,2,4]. If the operation includes caudal extension down to T3-T5, a second window can be created called the outside window (Figure 3) [2,4]. This can be accessed through retraction of the formerly mentioned vessels to the left, along with the right and left brachiocephalic vein [2,4]. The right brachiocephalic vein is retracted to the right, while the left brachiocephalic vein is retracted inferolaterally [2,4]. Through creation of the inside and outside windows, based on the brachiocephalic vessels, the cervicothoracic junction with caudal extension to T5 can be adequately exposed and accessed [1,2,4].

Nerves

When accessing the cervicothoracic vertebral bodies, certain nerves must be located to avoid over manipulation and damage to them in order to avoid complications. The recurrent laryngeal nerves must be located before retraction of the brachiocephalic vessels and the trachea when accessing the inside or outside widows [1,2,14,15]. On the right side, the recurrent laryngeal nerve lies more medial to the midline of the neck and can have a more variable connection to the vagus nerve [14,15]. Meanwhile, retraction of the trachea poses a risk of damaging the left recurrent laryngeal nerve [16]. Damage to either can lead to voice hoarseness due to paralysis of the vocal cords, dysphagia, and difficulty breathing [2,6,14,17]. Furthermore, the vagus nerve must be carefully retracted when exposing the vertebral bodies [2,14,18]. Careful retraction can avoid damage or stimulation, which can lead to interruptions in blood pressure and respiration intra-operatively [2,14,18].

Indications

**Figure 4:**
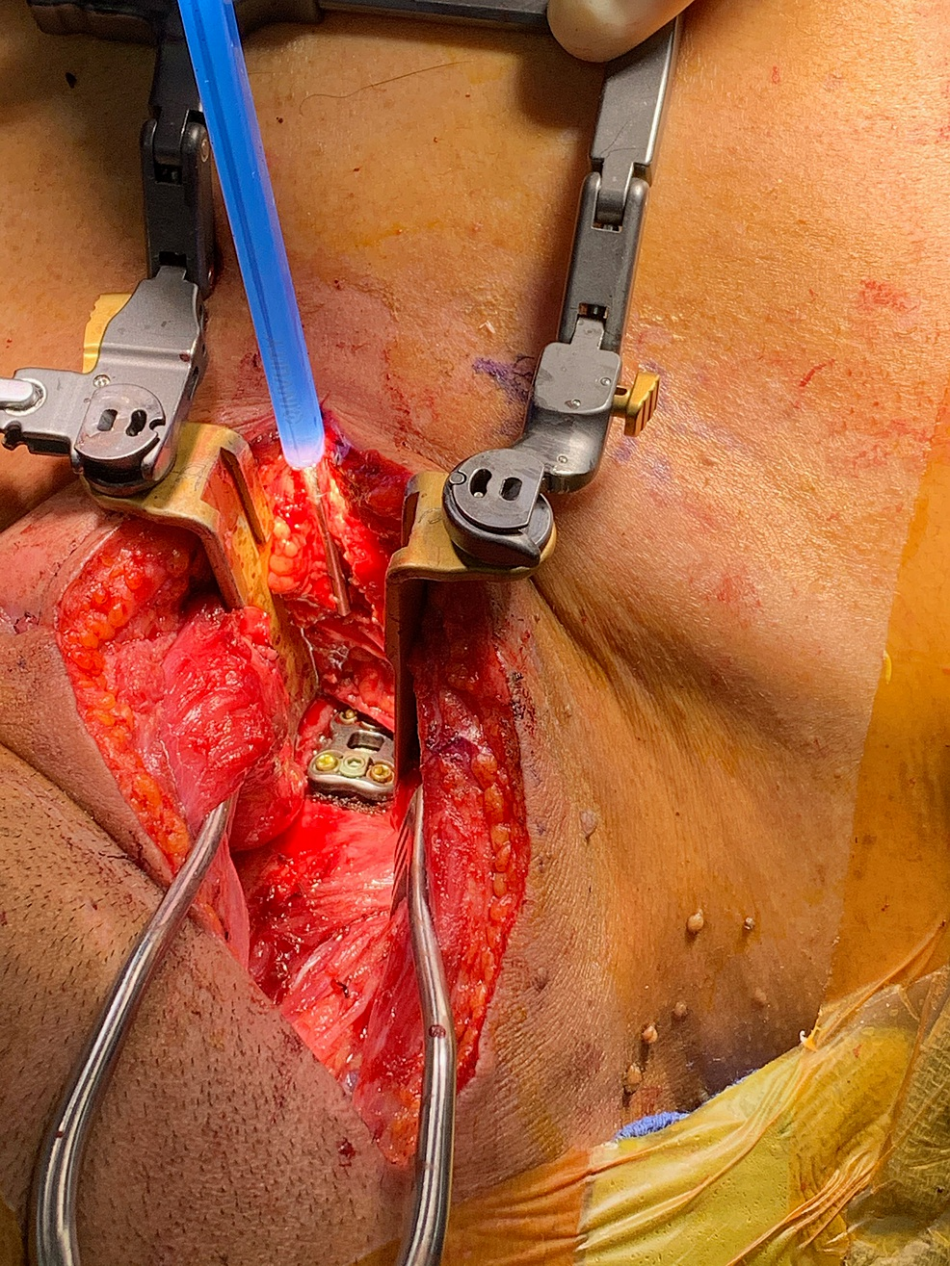
Exposure of the sternal-manubrial joint where a discectomy and interbody fusion was performed at the cervicothoracic junction.

The transsternal approach can utilize a full median sternotomy, but other modifications of this approach include manubriotomy with clavicle resection, partial lateral manubriotomy, and partial sternotomy with a transverse sternal split [19]. One indication for this approach includes tumors of primary or metastatic origin that are localized to the vertebral body or if an anterior decompression is indicated in addition or substitution to a posterior decompression. Additionally, other pathologies that may be treated through a transsternal approach are central or centrolateral calcified disc herniations causing neurological symptoms and pathological fracture-dislocation that results in the migration of bone fragments posteriorly (Figure 4) [4]. Infectious diseases such as tuberculosis affecting the upper thoracic vertebral bodies are another indication as demonstrated by Jiang et al. [2]. Finally, this approach may be used for the correction of cervicothoracic kyphosis with anterior spinal cord compression [18].

Contraindications

There are both spinal and non-spinal etiologies that contraindicate a sternotomy approach in spinal surgery. Non-spinal contraindications are typically cardiopulmonary in nature. This includes patients with severe lung disease such as chronic obstructive pulmonary disease (COPD) due to the risk of destabilization via the sternotomy approach, worsening the aforementioned conditions [20,21]. Further, patients with highly calcified aortic dilatation, decreased ventricular function, decreased ejection fraction, or morbid obesity are also relative contraindications - again due to the risk of destabilization [22]. In the spine, apical masses in close proximity to the phrenic or vagus nerves, the sympathetic chain, or the brachial plexus (particularly with concomitant symptoms of motor loss) contraindicate a sternotomy approach [23,24]. Although not an absolute contraindication, a previous median sternotomy typically contraindicates additional approaches due to increased risk of perioperative complications, including increased rates of morbidity and mortality directly attributable to redo-sternotomy [25,26].

## Discussion

Results and clinical outcomes

The sternotomy approach to the anterior spine has been used on a range of pathologies including Pott’s disease, primary and metastatic tumors, and spinal abscesses [1,2,6,14,15,27]. As early as 1957, reports have shown success in this approach to treatment [27]. Later attempts at this approach continued to show success while yet remaining experimental. In 1984, Sundaresan et al. showed a series of seven patients undergoing a sternotomy approach to the spine to treat spinal tumors and a pyogenic abscess [15]. All seven patients were considered to have improved in symptoms due to surgery; measured by pain relief, improvement in motor function and relief of any myelographic block [15]. Subsequently, it was reported that no patients suffered any sort neurological deficit as a result of the operation [15]. Later in 1986, eight patients undergoing an anterior approach to the spine for treatment of vertebral dislocations and spinal tumors showed success in this in this approach as well [14]. Only two cases of the eight showed minor injury during surgery, which included temporary paralysis and dysphagia due to injury to the recurrent laryngeal nerve [14].

In 2010, Zengming et al. showed terrific outcomes in a series of 54 patients undergoing a sternotomy approach to the spine [6]. Pathologies for the patients included spinal tuberculosis, metastatic tumors, eosinophilic granulomas and traumatic fractures [6]. The patient ages ranged from 37-69 years old, and the patients were followed for 24-48 months post-operatively. Pre-operatively, patients were evaluated and graded on the Frankel Grade classification and were re-evaluated post-operatively. It was reported that pain had resolved in all patients and many downgraded from higher Frankel grade classifications [6]. It was also shown that improvement in motor deficits was noted in those who presented with radiculopathy or myelopathy pre-operatively. Moreover, in those patients who underwent spinal fusion with autologous iliac bone grafts, successful fusion was shown in five to eight months. Furthermore, it was shown that there were no reported approach-related complications and no failure of any instrumentation used during the surgery. At follow-up, four patients were reported to have died; however, the cause of death was due to complications from systemic metastatic cancer [6].

In a separate series, (2010) Jiang et al. reported 16 patients with upper thoracic vertebral tuberculosis who underwent an anterior approach to the spine [2]. Among them, the patients suffered from intraspinal abscesses, paravertebral abscesses or both. The patients ages ranged from 37-72 years old and were initially evaluated on the Frankel grade classification. It was reported that all patients tolerated surgery well, and eight of them had downgraded on the Frankel grade classification and one patient showed no change in grade [2]. Eight patients were also reported to have had a pre-operative neurological deficit, and all showed improvement post-operatively. Lastly, spinal fusion was successfully reported at follow-up at three to six months [2]. Later reports in 2017, showed a case out of Ghana, West Africa of a 20-year-old male with spinal tuberculosis associated with vertebral osteomyelitis and cord compression who underwent a full median sternotomy to access the anterior spine with a successful clinical outcome [1]. The patient made a full recovery and radiographic images showed correction of cord compression [1]. Please refer to Table 1 for a chronological summary of clinical outcomes associated with an anterior approach to the spine.

**Table 1:** Summarization of selected publications on sternotomy access to the spine. N/A: not available

	Number of Patients	Diagnosis	Age Range	Results of Surgery	Improvement in Frankel Grade
Cauchoix and Binet, 1957 [27]	3	Spinal tuberculosis, spinal tumor (chondroma), abscess	4-29 years old	Improvement in neurological deficits	N/A
Sundaresan et al., 1984 [15]	7	Osteosarcoma, Ewing sarcoma, adenocarcinoma, breast malignancy, abscess	31-69 years old	Improved in pain, neurological deficit, and myelographic block	N/A
Lesion et al., 1986 [14]	8	Traumatic dislocation, spinal malignancy	Not reported	All cases considered successful	N/A
Zenming et al., 2010 [6]	54	Spinal tuberculosis, metastatic disease, eosinophilic granuloma, traumatic fracture	37-69 years old	Improvement in pain, neurological deficits, and successful spinal fusion	Yes
Jiang et al., 2010 [2]	16	Spinal tuberculosis	37-72 Years old	Improvement in neurological deficits and successful spinal fusion	Yes
Brogna et al., 2016 [4]	18	Spinal tuberculosis, disc herniation, metastatic disease, traumatic fracture, ankylosing spondylitis	33-53 years old	Improved neurological deficits, and successful spinal fusion	Yes
Okyere et al., 2017 [1]	1	Spinal tuberculosis	20 years old	Improved neurological deficits	N/A

Avoidance of complications

While sternotomy access to the spine has its advantages, it also comes with many risks that require a surgeon’s careful attention to detail. With this technique, there are many vital structures in close proximity to the incision including the carotid sheath, trachea, esophagus, recurrent laryngeal nerves, great vessels, vertebral arteries, and sympathetic trunk [19]. These vital structures should be correctly identified, tagged, and gently retracted by the thoracic surgeon before operating on the spine [19]. In addition, a meticulous dissection of the vascular compartment of the superior mediastinum by a thoracic surgeon is required to avoid injury to neurovascular structures [19]. Median sternotomy is associated with a relatively high risk of nerve injuries to the brachial plexus [28,29]. This is most likely due to accidental traction of the nerves, compression of the medial cord by the first rib, or fracture of the first rib [28]. Particular attention must be given to the brachial plexus to avoid such neurologic injury.

When beginning a sternotomy, it is important to identify the midline of the sternum for the proper point of incision [30]. Cutting the sternochondral cartilage or ribs is likely to lead to complications in repair or wound healing [31,32]. To avoid rupture of the brachiocephalic venous truncus or opening of the pleura, both major complication risks, the opening of the sternum must be done in slow progressive increments with a retractor [31,33]. Another common source of complication is incorrect placement of the drainage tubes at the end of the procedure. To avoid hepatic, epigastric, and colic lesions, the tubes should be placed under the fascia of the rectus muscle and avoid the epigastric peduncle [31]. When closing the pectoral fascia, subcutaneous tissues, and skin, careful repair of the linea alba is necessary to avoid incisional hernia [30,31]. Post sternotomy, patients are traditionally advised to avoid certain activities for four to seven weeks to ensure proper outcomes. These instructions are typically referred to as “sternal precautions,” and include avoiding activities such as lifting objects weighing 5-10 lbs and reaching behind the back [34]. However, more personalized and updated precautions have been proposed [35,36].

## Conclusions

The anterior cervicothoracic spine is particularly challenging to approach given the myriad of structures present in this region from vascular, osseous, nervous, and articular, lending to difficulties in exposure. As always, avoidance of complications is key. The sternotomy approach, although classically used in open-heart surgery has expanded to become a feasible and relatively safe approach in spinal surgery, which allows for a clear view of the anterior cervicothoracic vertebral bodies. This approach represents the most direct access to pathologies in the upper anterior cervicothoracic spine and enables the surgeon to gain direct exposure to the cervicothoracic junction. Based on our findings, the anterior transsternal approach is being utilized successfully in an increased number of cases demonstrating adequate safety with utility in treating various spinal pathologies from infective, metastatic, traumatic, and degenerative lesions. This technique is useful for the armamentarium of spinal surgeons. However, despite the purported advantages of this approach, anatomical considerations must be kept in mind while performing a sternotomy to prevent complications such as denervation or bleeding.
